# Broad spectrum of CRISPR-induced edits in an embryonic lethal gene

**DOI:** 10.1038/s41598-021-02627-y

**Published:** 2021-12-09

**Authors:** Kayla T. B. Fuselier, J. Michael Salbaum, Claudia Kappen

**Affiliations:** 1 Department of Developmental Biology, Pennington Biomedical Research Center/Louisiana State University System, 6400 Perkins Road, Baton Rouge, LA 70808 USA; 2 Department of Regulation of Gene Expression, Pennington Biomedical Research Center/Louisiana State University System, 6400 Perkins Road, Baton Rouge, LA 70808 USA; 3 Peggy M. Pennington Cole Chair in Developmental Biology, Pennington Biomedical Research Center/Louisiana State University System, 6400 Perkins Road, Baton Rouge, LA 70808 USA

**Keywords:** Genetic engineering, Biological techniques, Sequencing, Next-generation sequencing, Developmental biology, Embryogenesis, Gastrulation

## Abstract

Mendelian genetics poses practical limitations on the number of mutant genes that can be investigated simultaneously for their roles in embryonic development in the mouse. While CRISPR-based gene editing of multiple genes at once offers an attractive alternative strategy, subsequent breeding or establishment of permanent mouse lines will rapidly segregate the different mutant loci again. Direct phenotypic analysis of genomic edits in an embryonic lethal gene in F0 generation mice, or F0 mouse embryos, circumvents the need for breeding or establishment of mutant mouse lines. In the course of genotyping a large cohort of F0 CRISPants, where the embryonic lethal gene T/brachyury was targeted, we noted the presence of multiple CRISPR-induced modifications in individual embryos. Using long-read single-molecule Nanopore sequencing, we identified a wide variety of deletions, ranging up to 3 kb, that would not have been detected or scored as wildtype with commonly used genotyping methods that rely on subcloning and short-read or Sanger sequencing. Long-read sequencing results were crucial for accurate genotype–phenotype correlation in our F0 CRISPants. We thus demonstrate feasibility of screening manipulated F0 embryos for mid-gestation phenotypic consequences of CRISPR-induced mutations without requiring derivation of permanent mouse lines.

## Introduction

Neural tube defects (NTDs) are developmental abnormalities of neural tube closure, and are thought to have multifactorial origins in gene–gene and gene-environment interactions^1^. The neural tube is formed during the morphogenetic process known as gastrulation, and becomes closed at the end of neurulation. Disruptions in both of these processes are likely to cause embryonic defects, which -in severe cases- are lethal. In order to assess the contribution of candidate genes to the pathogenesis of NTDs in mouse embryos during gastrulation and neurulation, we employed a transient transgenic approach to mutagenize genes using the CRISPR/Cas9 system^2^. The approach is called transient, as the phenotype resulting from the genetic manipulation is analyzed directly in the F0 generation, without establishment of permanent mouse lines^3,4^. The advantage of such a strategy is that it allows for the screening of genes whose bi-allelic disruption may cause embryonic lethality, precluding the derivation of permanent mouse lines^5^. It has been estimated that 30% of murine null (knock-out) alleles are embryonic lethal in homozygous configuration^6^, the incidence of lethality in heterozygous configuration (haplolethal) is currently unknown.

Because each F0 gene-edited individual represents a unique genetic event, multiple individuals need to be generated and analyzed for morphogenetic defects to yield results that can be generalized over multiple distinct mutations by which the individuals might differ^7^, depending on accuracy of the CRISPR methodology at the given gene locus. While this can feasibly be achieved with transgenic mouse technology in two or three microinjection sessions per individual gene, the incidence and severity of phenotypes in the resulting genetically manipulated F0 progeny also allows estimates about the phenotypic penetrance of mutations at a chosen locus. In addition, a high-throughput transgenic approach enables the simultaneous targeting of multiple genetic loci^8^, thereby facilitating studies of gene–gene interactions during neural tube closure.

To establish feasibility of this methodology, we chose to mutagenize a gene whose function in mouse development has been well-characterized^9^, and whose mutant phenotypes can be visually ascertained at mid-gestation: the brachyury locus, which encodes the T-Box transcription factor T. Mice with a homozygous deletion of the T/brachyury gene are embryonic lethal by embryonic day 10, with defects in mesodermal derivatives, such as the notochord, presomitic and somitic mesoderm^10^. Reductions in T expression are associated with defective posterior mesoderm development, ranging from complete absence of posterior mesoderm^11^ to absent tail, abnormal somite development, short tail or curly tail, abnormal neural tube closure, kinked neural tube and spina bifida^12,13^.

We here report that similar morphological anomalies were observed in mouse embryos where the T/brachyury gene was edited by CRISPR mutagenesis, carried out in a transient transgenic strategy that involves phenotyping and genotyping of individuals directly in the F0 generation. It was only by employing single-molecule long-read sequencing, as implemented in the Oxford Nanopore sequencing technology^14^, that we were able to detect the multiple CRISPR-edited mutant T/brachyury alleles in single F0 individuals, and to identify larger on-target deletions than have been reported from previous CRISPR mutagenesis efforts.

## Results

**Figure 1:**
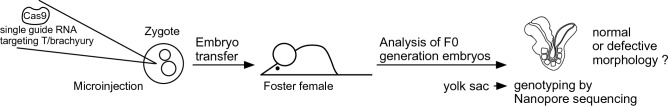
Transient transgenic strategy for CRISPR-induced mutagenesis. Schematic of the experimental approach: After microinjection of the Cas9/single-guideRNA ribonuclease complex into fertilized oocytes, the zygotes are transplanted into foster females. On gestational day E9.5, the morphology of individual F0 generation embryos is assessed, and the corresponding yolk sac tissue is collected for genotyping. This transient approach allows rapid screening of single, and potentially multiple, genes for developmental function without the need for breeding.

**Figure 2:**
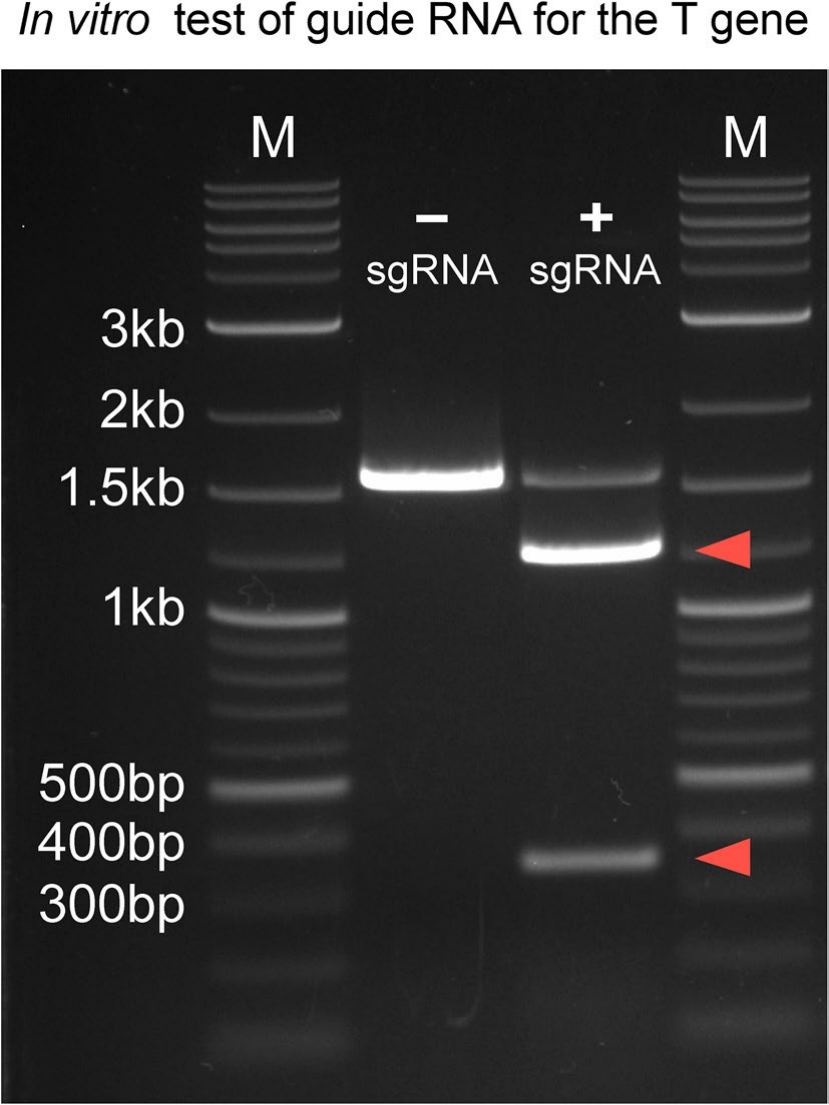
In vitro test of guide RNA for editing of the T gene. To detect CRISPR/Cas9-mediated cleavage at the intended targeted site in vitro, a 1592 bp region of the mouse T gene was PCR-amplified from genomic DNA and incubated with Cas9 protein and the single guide RNA (+ sgRNA). The control reaction contained the same substrate amplicon, and Cas9 protein, but no sgRNA (-sgRNA); the uncut PCR amplicon is close to 1.6 kb in size. Cleavage of the target amplicon by the sgRNA/Cas9 complex is detected by virtue of fragments of ~ 1250 bp and ~ 340 bp, respectively, in the sgRNA-containing sample (red arrowheads). M: Quick-Load® Purple 1 kb DNA Ladder (New England Biolabs, Ipswich, Massachusetts).

The principal experimental approach of this study is depicted in Fig. 1. The gene chosen for targeting encodes the T-box transcription factor T, mutations of which are responsible for the brachyury phenotype in mice^15,16^. The murine T gene consists of 8 exons and produces 3 known splice variants that are protein coding. We designed a guide RNA to target exon 3, as each of the known splice variants contain this highly conserved region. This exon codes for amino acid residues 158 through 202 of the T/brachyury protein, and encompasses a portion of the DNA binding-domain (amino acid residues 1–229). Thus, disruption of the third exon is expected to abolish transcription factor function. The efficacy of the specific guide RNA was ascertained in vitro, as shown in Fig. 2.

**Figure 3:**
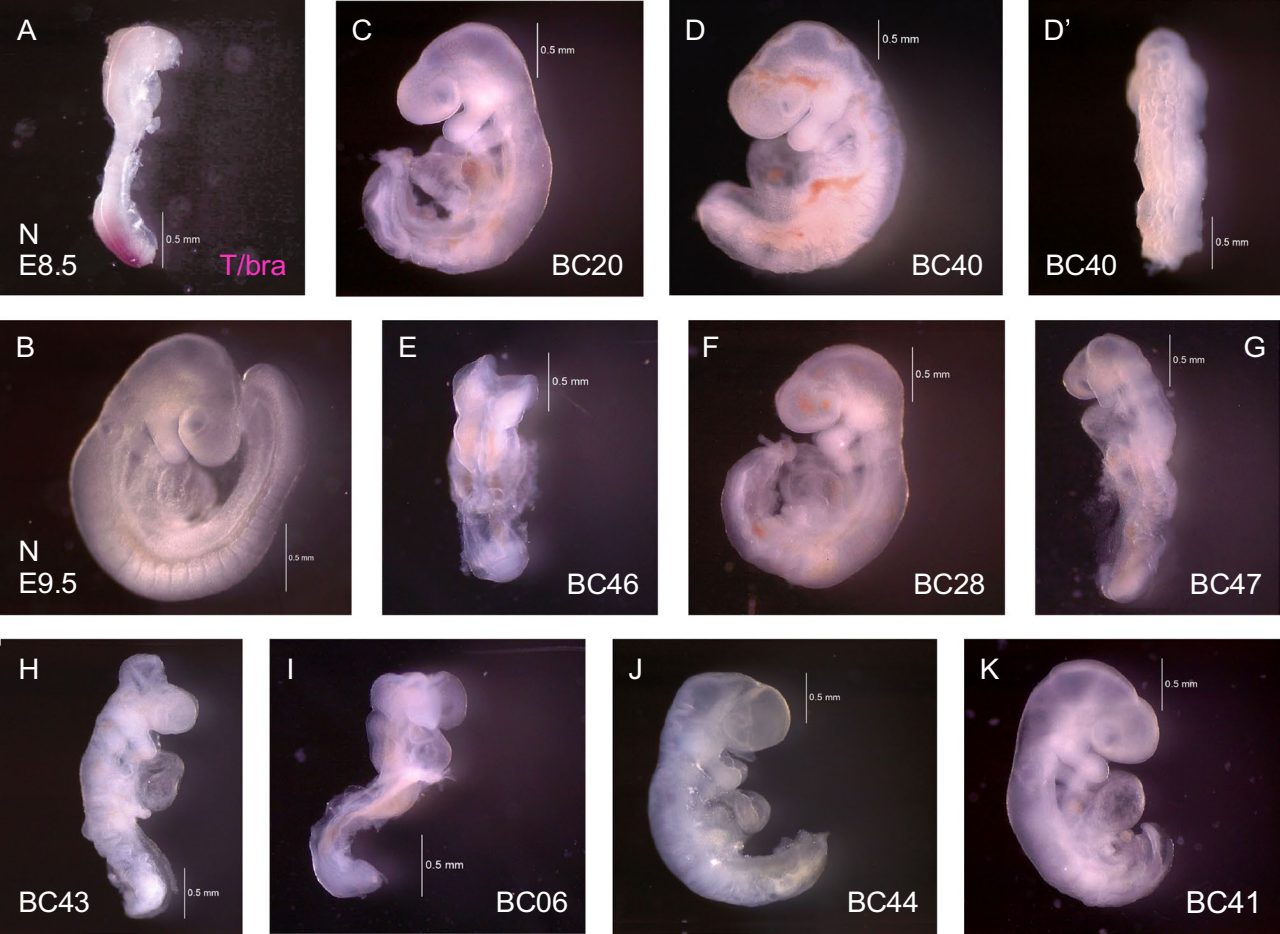
Morphological abnormalities of individual CRISPants. Panels A and B show normal mouse embryos at E8.5 (**A**) and E9.5 (**B**). Based upon the earlier expression of T/brachyury in the primitive streak, notochord, and the posterior region (as shown by in in situ hybridization in (**A**), disruption of the coding region by targeting the third exon for CRISPR editing is expected to cause morphological abnormalities in somite formation, neural tube morphogenesis and reduced growth of the posterior. Panel B shows a normal mouse embryo with well-formed somite boundaries, and long posterior region. CRISPants are designated according to their barcode numbers (see Table 1), and they are ordered in this figure by ascending content of mutant alleles. BC20 and BC40 (**C**, **D**, and **D′**) carry only the ∆11@-1 ϴ allele, likely in homozygous configuration, and they exhibit disrupted somite formation and an abnormal tail region, accompanied in BC40 by wavy neural tube (**D′**). (**E**) BC46 carries the ∆11@-5 ϴ allele, and displays a disordered neural tube, as well as a somite-less and shortened posterior region. (**F**) BC28 contains both ∆11@-1 ϴ and ∆11@-5 ϴ deletions; while the neural tube has closed in the anterior region, somites are not discernable in the trunk and shortened posterior region, which also reveals a wavy neural tube. (**G**) BC47 harbors the longest 2728 bp and both 11 bp deletion alleles; the neural tube is disordered and wavy in the trunk, heart development is compromised and somites are absent in the posterior. (**H**) BC43 carries 3 deletions ranging from 719 to 998 bps, a 10 bp deletion and the one nucleotide insertion alleles; morphogenesis of somites and neural tube is abnormal, with wavy neural tube and shortened tail region. (**I**) BC06 contains 3 deletions over 1000 bps in size, and 691 bp and 100 bp deletion alleles; the entire trunk region is malformed and shorter, somites are absent, and heart development is also abnormal. (**J**) BC44 harbors 6 deletion alleles, and exhibits lack of somites, wavy neural tube from the hindbrain along the entire trunk, and a severely truncated tail region. (**K**) BC41 carries 6 different deletion alleles, and displays complete absence of somites and neural tube below the heart and in the posterior trunk.

We injected fertilized oocytes with Cas9 enzyme and a single-guide RNA targeted to the T/brachyury gene; these manipulated zygotes were transplanted into pseudopregnant foster females and allowed to develop until gestational day E9.5. Embryos were harvested, and the yolk sac associated with each individual was used for genotyping of the targeted region. Morphological analysis of embryos revealed a spectrum of phenotypes (representative examples are shown in Fig. 3), encompassing defects that have been described in the literature for various heterozygous and homozygous configurations of T-alleles in mutant mouse strains: wavy and kinked neural tubes, defective neural tube closure, malformed somites, short tails, absent tails, malformed posterior and trunk regions^10,13,17,18^. All recovered embryos displayed one or more of these morphogenetic abnormalities. Notably, we found considerable phenotypic variability between individual F0 embryos, suggesting that the underlying gene editing events may also vary between gene-edited individuals, which we refer to as CRISPants.

**Figure 4:**
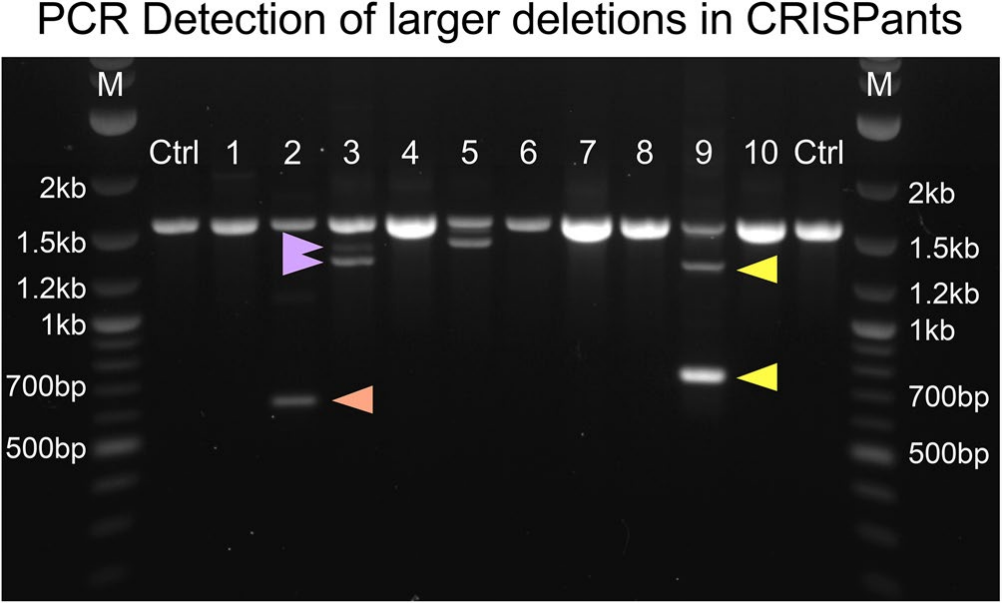
PCR amplification of genomic DNA reveals larger deletions. A 1592 bp region spanning the third exon of the T gene was amplified from genomic DNA of 10 individual CRISPants (numbered 1 through 10) and genomic DNA from unmanipulated mice (Ctrl: control). The orange triangle identifies an amplicon of approximately 650 pb length in sample 2, indicative of a large deletion with an estimated length of 950 bp. Purple arrows point to bands in sample 3 of ~ 1.4 and ~ 1.3 kb size, respectively, indicating putative deletions of ~ 200 and ~ 300 bp size. Yellow arrows mark amplicons in sample 9 of ~ 1.3 kb and ~ 800 bp size, predicting the presence of deletions of ~ 300 bp and ~ 800 bp, respectively. M: Lane of marker DNA fragments of indicated sizes.

In order to genotype the putative edited target region in the T/brachyury gene CRISPants, we initially planned to employ a widely used combination of PCR-based amplification, followed by subcloning and DNA sequencing. PCR amplification of the targeted region was expected to produce amplicons of ~ 1.6 kb from genomic DNA (see Fig. 4, Ctrl samples). Unexpectedly, however, visualization of the PCR products after gel electrophoresis revealed the presence of much smaller than expected amplicon sizes in several of the samples, and often multiple smaller bands within a single sample (see Fig. 4 for an/example/s). We interpreted the smaller products to reflect larger-than-expected deletions, and the multitude of bands to reflect the presence of multiple events within a single CRISPant. For example, 3 bands of approximate sizes of 0.8 kb, 1.3 kb and 1.6 kb are detected in sample #9 (Fig. 4), suggesting that deletions of potentially 0.8 kb and 0.3 kb could be present. In order to circumvent the laborious steps of subcloning for separation of distinct molecular species in the amplicon mixture, and subsequent sequencing of numerous clones for each sample/individual, we employed single-molecule long-read sequencing of amplicons from the target region, as afforded by the Nanopore sequencing approach.

Again, PCR was employed to amplify the region of interest, this time increasing the amplicon length to 6.6 kb around the targeted cut site ϴ (theta), and used to prepare Nanopore sequencing libraries. Sequences were aligned to wildtype T locus control samples run alongside the experimental samples, and to the reference genome sequence. The software pipeline CRISPResso2 was used to visualize the sequence alignments and insertion or deletion (INDEL) events. Position of INDEL events were labelled in accordance to their distance from ϴ, with negative values on the 5’ left side, and positive values designating the 3’ right side. A complete listing of the 140 editing events observed amongst 40 CRISPants for T/brachyury is presented in Table 1.

**Table 1:** Summary of Nanopore sequencing results for independent CRISPants.

Sample	Barcode	Alleles according to CRISPResso analysis												Wildtype in %	# of deletions	# of insertions	Total # of INDELs
		INDEL 1	in %	INDEL 2	in %	INDEL 3	in %	INDEL 4	in %	INDEL 5	in %	INDEL 6	in %				
S01M01A	BC05	+1bp@1	35.88	−16bp@−3	23.12	−530bp@−217	17.62	−419bp@−104	3.16					20.21	3	1	4
S01M01B	BC06	−1021bp@−1007	55.32	−1004bp@−995	14.88	−100bp@−6	13.03	−1006@−999	11.45	−691@−676	3.65			1.67	5	0	5
S01M01D	BC07	−320bp@−285	29.78	−11bp@−1	26.64	−130bp@−11	19.40							24.19	3	0	3
S01M01E	BC08	+1bp@1	43.15	−6bp@−2	27.34	−1460bp@−2	6.55	−13bp@1	4.07					18.90	3	1	4
S01M01F	BC09	−149bp@−3	30.56	−6bp@−2	25.38	−44bp@−29	17.82	−11bp@−5	14.33	−109bp−94	5.70			6.21	5	0	5
S01M08A	BC10	−11bp@−5	36.12	+1bp@1	32.25	−23bp@−15	10.09							21.54	2	1	3
S01M08B	BC11	−607bp@1	44.97	−11bp@−1	14.76	−982bp@−965	7.94	−435bp@1	4.70					27.63	4	0	4
S01M08D	BC12	−19bp@−14	34.76	−10bp@−8	32.16	−6bp@−2	15.90							17.19	3	0	3
S01M08E	BC13	−11bp@−5	27.41	−11bp@−1	20.68	−3bp@1	20.95	−1bp@1	20.49					10.47	4	0	4
S01M08F	BC14	−153bp@−148	34.30	−10bp@−4	23.43	− 1bp@1	30.75							11.52	3	0	3
S01M08G	BC15	−6bp@−2	25.25	+1bp@1	23.61	−11bp@−5	18.13	−11bp@−1	17.46					15.55	3	1	4
S01M08H	BC16	−2bp@1	52.54	−11@−5	28.24	−11@−1	10.44							8.78	3	0	3
S01M08I	BC17	−332bp@−2	48.07	−11bp@−5	33.31	−22bp@−11	8.19	−11bp@−1	7.42					3.00	4	0	4
S01M08J	BC46	−11bp@−5	67.24											32.76	1	0	1
S01M08L	BC47	−2728bp@−386	93.58	−11bp@−5	3.06	−11bp@−1	2.40							0.96	3	0	3
S01M10A	BC20	−11bp@1	94.17											5.83	1	0	1
S01M10B	BC21	−659bp@−174	60.99	−510bp@−3	38.98									0.03	2	0	2
S01M10C	BC22	−972bp@−504	47.43	+1bp@1	23.26	−227bp@−218	16.09							13.22	2	1	3
S01M10D	BC23	−11bp@−1	42.77	−241bp@−1	25.85	−11bp@−5	25.29							6.09	3	0	3
S01M10E	BC24	−496bp@−487	37.46	−412bp@−204	14.13	−11bp@−5	9.63	−498@−484	8.39					30.39	4	0	4
S01M18A	BC25	−11bp@−5	40.62	−4bp@−3w/sub@−4(C)	37.26	−346bp@−337	9.22	−34bp@−30	6.63					6.27	4	0	4
S01M18B	BC26	−11bp@−5	44.65	−29bp@−21	28.03	−142bp@−3	20.99							6.33	3	0	3
S01M18C	BC27	−23bp@−15	55.70	−11bp@−5	18.50	+1bp@1	16.99							8.81	2	1	3
S01M18D	BC28	−11bp@−5	49.40	−11bp@−1	43.28									7.32	2	0	2
S01M20A	BC29	−471bp@−3	33.27	−192bp@−12	27.10	−11bp@−5	16.93	−295bp@−12	13.78	−20bp@−7	6.56			2.35	5	0	5
S01M20B	BC30	−453bp@−2	27.79	−2bp@−1w/2subs@−3,−4(GA)	22.56	−1bp@1	18.38	−11bp@−1	13.41					17.86	4	0	4
S01M20C	BC31	−11bp@−5	31.45	−908@−160	23.90	+1bp@1	23.37	−11bp@−1	6.67					14.61	3	1	4
S01M20D	BC32	+1bp@1	50.70	−11bp@−5	23.95									25.35	1	1	2
S01M20E	BC33	−746bp@−2	41.41	−13bp@−13	25.70	−8bp@−1,w/2subs@8,9(AT)	14.93	−11bp@−1	13.50					4.47	4	0	4
S01M20G	BC34	−11bp@−1	40.00	+1bp@1	38.32									21.68	1	1	2
S01M20H	BC35	−11bp@−1	37.25	−18bp@−8w/2subs@−13,−12(CG)	36.32	−88bp@−3	10.39	−284bp@−6	8.38					7.66	4	0	4
S01M20J	BC36	−782bp@−9	62.20	−11bp@−5	13.81	−72bp@−9	8.31							15.68	3	0	3
S01M20K	BC37	−670bp@−656	34.28	−482bp@−3	32.86	−169bp@−133	21.52	−11bp@−5	9.90					1.44	4	0	4
M1 B	BC38	−912bp@−3	43.05	−317bp@−6	20.32	−11bp@−1	18.03	−6pb@−2	16.49					2.11	4	0	4
M1 C	BC39	−2451bp@−310	53.53	−11bp@−1	22.93	−11bp@−5	9.94	−6bp@−2	10.67					2.93	4	0	4
M1 E	BC40	−11bp@−1	96.64											3.36	1	0	1
M8 A	BC41	−936@−931	32.17	−11@−1	20.01	−643@−3	14.45	−477@−449	11.88	−208@−15	10.54	−24@−12	9.64	1.31	6	0	6
M8 B	BC42	−859bp@−846	52.34	−6bp@−2	21.81	−295@−4	13.90	−11@−1	9.14					2.81	4	0	4
M8 D	BC43	−719bp@−709	30.03	−998bp@−989	21.36	−10bp@−4	21.67	+1bp@1	12.33	−738bp@−725	2.32			12.29	4	1	5
M8 E	BC44	−817@−6	23.90	−1bp@1	20.45	−11@−1	19.63	−373@1w/sub@−1(T)	13.10	−11bp@−5	7.63	−101@−1	5.52	9.77	6	0	6

Sequence analysis was performed with CRISPResso2. Alleles are identified by their INDEL size (deletions as −, insertion as +; position is given relative to ϴ), ordered by most frequently appearing allele sequence. A few deletions contained substitutions, as noted in parentheses. After identification of all alleles present in a sample, the prevalence for each allele was determined as the % of sequence reads aligning with that particular allele.

**Figure 5:**
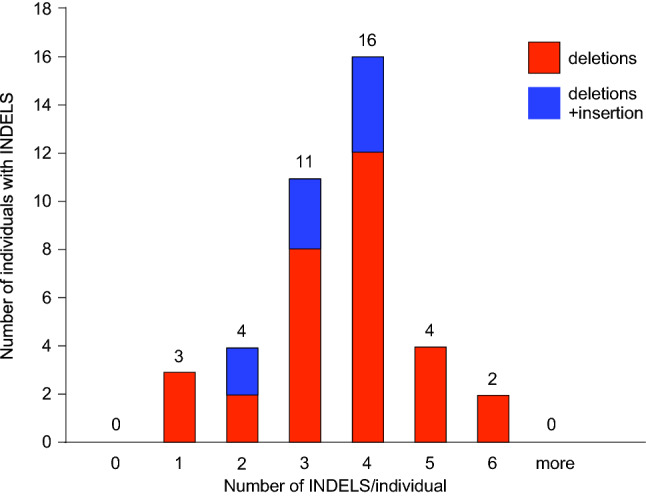
Occurrence of INDELS in individual CRISPants. The number of mutant alleles per individual was plotted. Red: deletions, Blue: insertions. 33 CRISPants contain three or more mutant alleles of the T/brachyury locus.

**Figure 6:**
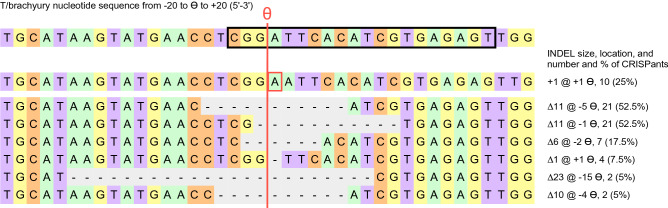
INDELS with occurrence in more than one individual. The sequence around ϴ is shown, for 20 bp on either side. The black box frames the sequence complementary to the targeting portion of the single-guide RNA. The position of the cut site ϴ is indicated by the vertical red line. Only one insertion was detected, the addition of an A, which was present in 10 independent CRISPants. Deletions that were found in 2 or more individuals are listed in descending order of frequency; 10 individual CRISPants had both 11 bp deletions.

These 140 editing events represent predominantly deletions (∆), ranging from 1 to 2728 bps in size. Most commonly observed were 4 editing events per T CRISPant (in 16 of 40 embryos), with some CRISPants harboring fewer, or more -up to 6-distinct mutations (Fig. 5, and Table 1). The only insertion detected was a 1 bp insertion at position + 1 relative to ϴ (Fig. 6), which occurred in 10 (25%) of the CRISPants. All other events were deletions.

Of the 130 deletion events, 80 unique alleles could be recognized; of those, the 6 deletion events that occurred in multiple embryos are shown in Fig. 6. The two most common deletions were 11 bps in size, and started at either −5 relative to ϴ (in 21 of 40 CRISPants; 52.5%) or at −1 ϴ (also in 21 of 40 CRISPant; 52.5%), respectively. Eleven of our T CRISPants had only one of these 11 bp deletions, respectively, and 10 T CRISPants harbored both (32 embryos with one or both of the recurrent 11 base deletion at −5 ϴ or −1 ϴ). Additional deletions observed in more than one embryo were 23 bp or 10 bp in size (each found in 5% of CRISPants), 6 bp (in 17.5% of CRISPants), and one base pair deletions in 4 CRISPants (7.5%).

**Figure 7:**
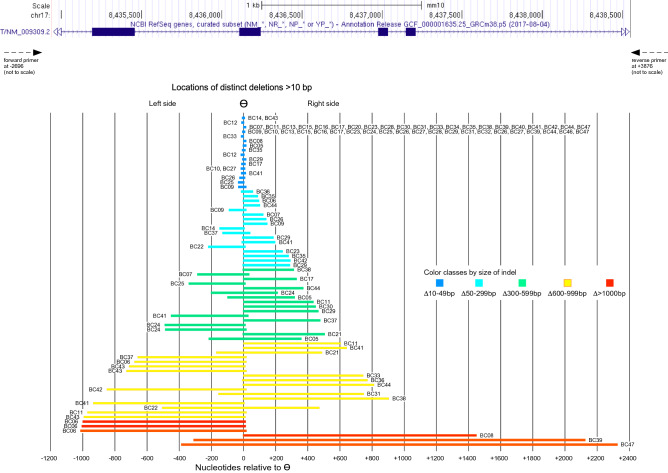
Locations of individual larger deletions in the T locus of CRISPants. Each deletion of greater than 10 bp is shown as a single line, ordered by overall size from the bottom up. All deletions end at, originate at or span across ϴ. Of note, a large fraction of the deletions are asymmetric relative to the cut site. Deletions are colored according to size, and the individual samples in which they appear are listed next to the respective line.

Consistent with the results of the initial PCR analysis (Fig. 4), we also detected much larger deletions, ranging up to 2728 bps in size (see Table 1). A schematic of all deletions longer than 10 bps is shown in Fig. 7. Notably, although the longer deletions all spanned or involved the targeted cut site ϴ, many were located asymmetrically, with 25 deletions located to a much greater extent towards the 5′ side of ϴ compared to the 33 deletions located to a much greater extent towards the 3′ side. Thus, deletions predominantly "spread" in either of the two directions from the cut site, and "spreading" evenly in both directions is observed mostly to involve fewer than 10 nucleotides on either side. There were 46 unique "start sites" for deletions, including θ itself (in 7 distinct events); 56 distinct "end sites" were observed.

The consequences of the CRISPR-induced mutations render the T/brachyury protein dysfunctional. The targeted Exon 3 encodes 72 amino acids of the N-terminal DNA-binding domain, contributing residues 159–230 to the protein, which contains 436 amino acids overall. Multiple protein changes would be predicted from the CRISPR-mediated double strand breaks and repair. For examples, the 1 bp insertion at ϴ creates a frame shift, changing 31 amino acids before a premature stop codon at position 201. The 11 bp deletion at −5 relative to ϴ also shifts the frame, changing 29 amino acids before a premature stop codon at position 197. The 11 bp deletion at −1 ϴ changes 27 amino acids before a premature stop codon. The 1 bp deletion at +1 ϴ introduces a frame shift, changing 3 amino acids before a premature stop codon at position 172. While the 6 bp deletion at −2 ϴ causes an in-frame loss of just 2 amino acids, those are located within the highly conserved DNA binding site. Obviously, the larger deletions will remove larger parts of the protein, critically altering protein structure and affecting DNA-binding function. In addition, deletions that extend more than 40 bp in the 5′ direction of ϴ will also remove the splice acceptor site at the 5′ end of Exon 3, and deletions larger than 210 bp to the 3′ of ϴ will result in loss of the Exon 3 downstream splice donor site; both events, alone and in combination in the same allele, will alter the structure and length of any potential transcript, and of the protein produced, if any. As reduced expression as well as reduced activity of the T transcription factor is known to cause developmental defects in established genetic models, the CRISPR-induced mutations we detected in the F0 generation can explain the phenotypic alterations in all of the CRISPant embryos we recovered.

**Figure 8:**
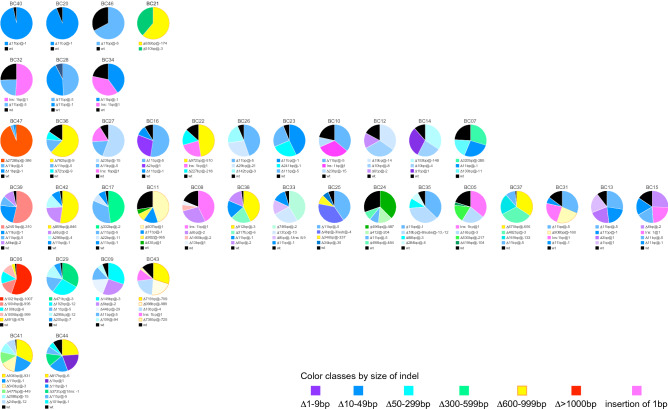
Representation of T/brachyury allele sequences within each individual. Colored segments depict the fraction of each edited allele among all sequences for the respective sample; wildtype sequences are indicated by black color. The color scheme corresponds to the same deletion size classes as defined in Fig. 7. Size of each INDEL (as listed in Table 1) is given in the legend below each sample. Samples are ordered by the total number of alleles detected (edited and wildtype), with individuals harboring 2 alleles at the top, and those with the most alleles at the bottom of the figure. Within each allele class, samples are ordered from left to right according to descending proportion of the most prominent edited allele.

Intriguingly, different CRISPR-edited alleles within the same individual were found to be represented in different fractions of all sequences for the individual, ranging from as much as 96.6% for the predominant allele in BC40 to as little as 2.32% for the minor allele in BC43, and wildtype sequence representation was found to range from 0.03% in BC21 to 32.8% in BC46 (Table 1). The proportions of alleles and their number in each individual sample are depicted in Fig. 8 as segments of pies. It is visually obvious that all CRISPants have unique genotype configurations. Minor fractions of alleles could conceivably be due to low cell numbers with the particular allele configuration within the genotyped tissue (here yolk sac), while more prominently represented alleles likely reflect (a) more predominant cell lineage(s). Due to the complexity of genetic configurations within any given individual and the low number of individuals with the smallest or largest numbers of mutant alleles, we were unable to establish an unequivocal correlation between number of mutant alleles and phenotype severity, or particular mutant alleles and phenotype severity. However, all recovered CRISPants exhibited morphological anomalies consistent with on-target editing of the T/brachyury gene, demonstrating high phenotypic penetrance of the CRISPR-edited mutant alleles.

## Discussion

Our goal in this work was to test the feasibility of a transient approach to identify new genes that—alone or in combination—contribute to morphogenesis and closure of the neural tube. Because neural tube defects originate early in post-implantation development, during the processes of gastrulation and neurulation, and because they can cause postnatal lethality, any genetic manipulation that targets this process may impact postnatal survival and preclude establishment of a live mutant strain. Therefore, we needed to screen for morphological defects in gene-edited embryos directly in the F0 generation, prior to birth. We chose to target a gene for which morphological phenotypes can be detected with ease, and at high penetrance under conditions of homo- as well as hetero-zygosity at the targeted locus, the gene encoding the T/brachyury transcription factor. Our results demonstrate that CRISPR-induced edits occurred in every individual recovered, and that they caused phenotypic anomalies that resemble those observed in conventional genetic mutations at the T/brachyury locus^11–13,17,18^, including posterior growth defects, somite defects, wavy neural tube, open neural tube.

Historically, the transgenic mouse field operated under the convention that a minimum of three independent genetic events yielding the same outcome would be required to establish that the observed phenotypic outcome was caused by the genetic manipulation^19^, as opposed to some random parameter, which would not be expected to occur consistently among multiple individuals. However, in cases of incomplete penetrance or phenotype variability (also termed variable expressivity), larger sample numbers are necessary to document the phenotypic spectrum with convincing robustness. In the absence of prior evidence from, e.g. targeted mutant mouse lines, the optimal sample number can only be determined post-hoc. Phenotypic variability for T/brachyury mutant mouse lines has been mentioned in the literature (although not quantified), but those reports used strains with genetic backgrounds different from FVB/N. In our CRISPR-mediated editing of the T/brachyury gene, 39 embryos from multiple pregnancies and different microinjection sessions were recovered, and they all exhibited malformations previously reported from multiple T-mutant strains^10,13,17,18^. Thus, in recapitulating developmental defects with 100% penetrance within the known phenotypic spectrum, our work provides proof-of-concept for CRISPR-induced gene editing as a feasible strategy to assess gene function in embryonic development without the need for the establishment of permanent mouse lines.

By employing single-molecule long-read sequencing, we detected numerous distinct CRISPR-induced deletion events that involved longer regions of the targeted gene than initially expected. A large fraction of deletions was asymmetric relative to the cut site, a phenomenon that was also observed by others^20^. Notably, the largest deletion we found was close to 3 kb in size. We would not have detected the larger deletions if we had followed the genotyping approaches that were standard in the field at the beginning of our project: Published protocols recommend sequencing of considerably shorter amplicons, such as for example in the 100–200 bp size range^21^. Under those criteria, (assuming sequencing spanned across ϴ by 100 bp on either side), only 20 individuals out of our 40 CRISPants would have genotyped as CRISPR edited, and 54 of the 80 unique deletion events would have been missed. Similarly, following recommendations for amplification and sequence analysis included in the widely used Webtool CHOPCHOP (150–290 bp amplicons^22,23^) or the *CRISPR Gene Editing* guide (recommending sequencing of 500–1500 bp, starting 200 bp upstream of ϴ^24^), we would have detected only 30 out of the 81 unique INDEL events, and missed 23 unique deletions, respectively, scoring as successfully edited only 12 or 26 of our 40 CRISPants, respectively. Such low genotyping ascertainment would have been difficult to reconcile with the frequency of morphological defects seen in all of our CRISPants. Thus, the choice of long-read sequencing technology not only enabled us to identify genome edits in every recovered embryo, but was critical to our success in attributing abnormal phenotypes to underlying mutations.

Another advantage of the Nanopore MinION sequencer is the large number of reads that can be acquired, mitigating the inherently higher error rate of the technique by creating reliable consensus sequences^25^. This is particularly useful for detection of CRISPR-mutagenesis in mouse strains or animal species for which no reference genome sequence exists^26,27^. The largest deletion we observed encompasses 2728 base pairs, and we would have been able to recognize larger events, as long as they fell within the 6.6 kb size of our PCR amplicons. However, we cannot exclude the theoretical possibility that rare events extending beyond the ends of the amplified product could be present in our CRISPants. For CRISPR transgenic approaches where larger amounts of genomic DNA are available than can be prepared than from yolk sac, such as for example when genotyping delivered F0 or F1 generation progeny, direct Nanopore sequencing of genomic DNA could potentially allow identification of even larger deletions, or structural alterations^28^, and therefore be an attractive alternative to amplification- or cloning-based genotyping.

Our genotyping results demonstrate that we efficiently generated genome edits in every embryo recovered after microinjection and implantation. While we recovered 2 CRISPants with a single mutation and 4 CRISPants with just two mutations, it was the availability of a large collection of single-read sequences that enabled unequivocal identification of multiple mutant alleles in single individuals. The majority of CRISPants in our study harbored three or more mutant alleles, indicating that these latter individuals each were a mosaic of at least two (or more) genetically distinguishable cell populations. The extent to which cells with different genetic configurations are apportioned to trophoblast layer or the inner cell mass^29^, which generates the embryo proper, is likely variable between CRISPants, and may account for the differential prevalence of particular alleles within an individual embryo. If live progeny were derived and bred to subsequent generations, only alleles that are present in germline cells would be transmitted to the next generation^7^. In addition, unequal representation of alleles among the sequences for a given CRISPant could also be a reflection of the mixed cell composition of the yolk sac tissue that was the source of genomic DNA for genotyping. The yolk sac consists of two layers: a visceral endoderm layer derived from the primitive endoderm and an extra-embryonic mesoderm layer derived from epiblast cells that also generate tissues of the embryo proper^30^. However, we cannot exclude the possibility that the embryo proper could harbor additional mutant alleles that are not represented in yolk sac cells.

**Figure 9:**
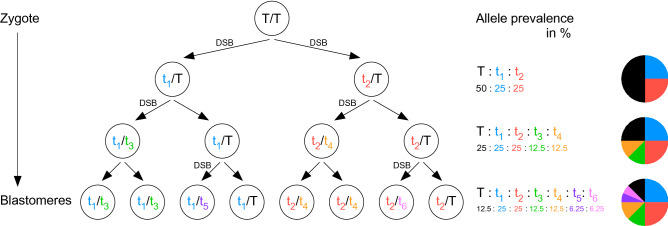
Successive CRISPR-induced mutations during the first embryonic cell divisions. Depicted is the most parsimonious theoretically possible scenario by which 6 mutant alleles in the same individual can be induced. T: wildtype allele, t_1–6_: different mutant alleles (separate colors). As cells divide, DNA strand breaks (DSB) can occur on either sister chromatid, with retention of wildtype allele on the intact strand. The different alleles will be represented at different prevalence among all cells in successive generations, as visualized in the pie diagrams (colors correspond to the respective mutant alleles t_1–6_).

In a given individual, up to 6 distinct mutant alleles were detected (see Table 1 and Fig. 8), in addition to low or appreciable fractions of wildtype sequence. Very minor fractions of wildtype sequence could be attributable to potential contamination during dissection with wildtype cells from the surrogate mother, such as blood or uterine decidual cells. But greater proportions of wildtype sequences suggest that a fraction of cells in an individual could be heterozygous for a CRISPR-induced mutation, due to incomplete editing at the single-cell stage. Yet, the large number of mutant alleles detected in 40 individual samples, with the majority of individuals harboring over 3 mutant alleles, and the fact that not a single animal with wholly unmodified genome was recovered, taken together indicate that CRISPR-mediated editing can proceed from the zygote through subsequent cell divisions. The most parsimonious explanation for the preponderance of more than 3 mutant alleles in our CRISPants is that editing occurred—at the least—during the cell divisions that produced the 2-cell and 4-cell stages; for the rarer occurrence of 5 and 6 mutant alleles in the same individual, editing would have to have continued at least through the 8-cell stage (Fig. 9).

If wildtype alleles are retained through one or more cell divisions, the multiple alleles in individuals are predicted to be present in unequal proportions even after the first three cell divisions (Fig. 9), which is consistent with our results (Fig. 8). In principle, differential representation of alleles could also be due to reduced efficiency of longer amplicon production in the PCR, but then one would expect that longer deletions be preferentially overrepresented, owing to shorter amplicon size. Instead, we find CRISPants with either of those scenarios: both large or small deletions can predominate in a given individual: For examples, in CRISPants BC06 and BC47 large deletions predominate, in CRISPants BC08 and BC31 small deletions are the most prominent allele, respectively (Table 1 and Fig. 8), arguing against technical artifact. Therefore, we attribute the differential representation of INDELs in individuals with multiple alleles to sequential editing events through successive cell divisions. Given that the majority (33 of 40) of our CRISPants harbors more than 3 distinct mutant alleles, the most plausible scenario is that most of their cells would be compound heterozygous for two different INDEL events.

In summary, employing single-molecule sequencing enabled us to identify numerous mutant alleles of the T/brachyury locus, a large fraction of which would have been missed when genotyping with the more commonly used shorter-read sequencing approaches. Furthermore, our study in T/brachyury-targeted CRISPants provides proof-of-concept for the feasibility of screening manipulated F0 embryos for phenotypic consequences of CRISPR-induced mutations in genes where disruption of one or both alleles might cause embryonic lethality. The particular promise of this strategy is that it now allows simultaneous targeting of combinations of two or more genes with suspected functions in early embryonic development, and especially in morphogenesis of the neural tube.

## Methods

### Design of single guide RNA and in vitro testing for activity at the T/brachyury locus

The CRISPR single-guide RNA (sgRNA) was designed to target an essential coding region^31^ contained within all known isoforms of the T/brachyury gene mRNA, and its suitability was assessed with the online tool at crispr.mit.edu (https://zlab.bio/guide-design-resources). The targeting RNA sequence was selected with as few predicted off-target events as possible, and yielded a score of 88 (on a scape of 1–100 where higher is better) in the MIT Specificity test^32^ (ACTCTCACGATGTGAATCCG; NCBI location #17:8,436,143–8,436,163). In addition, the whole single-guide RNA used in this study contained the canonical PAM, CRISPR RNA [crRNA] and tracrRNA sequences for the Cas9 complex; it was purchased from Invitrogen (https://www.thermofisher.com/us/en/home/life-science/genome-editing/geneart-crispr/crispr-libraries/trueguide-grnas.html). Re-analysis with the currently available tools CRISPOR^33^, CRISPick^34,35^, and CHOPCHOP v3^23^ yielded the highest ranking for our guide RNA in all three algorithms.

DNA fragments PRC-amplified from FVB mouse genomic DNA were used to test the sgRNAs for activity in vitro. A 1592 bp segment of the T/brachyury gene was amplified by employing the following primers: T-Forward TGAGGAGGAAGGAGGCATTC (ENSEMBL Gene ID: ENSMUSG00000062327, Chromosome 17:8,434,905–8,434,925, GRCm38.p6) and T-Reverse GCTGGCGTTATGACTCACAG (ENSEMBL Gene ID: ENSMUSG00000062327, Chromosome 17: 8,436,477–8,436,497). PCR conditions consisted of 50 μl reactions, with 50 ng of template, 200 nM primers, 250 μM each dNTP and 1.25 U Taq DNA Polymerase (New England BioLabs). Amplification conditions employed Hotstart, with 2 min at 95 °C, followed by 30 cycles of: 30 s melting at 95 °C, 30 s annealing at 59 °C, 3 min elongation at 68 °C, and a final step of 10 min at 68 °C, after which the samples were kept at 4 °C.

The amplicon produced contained the intended target cut site Theta (ϴ) in asymmetrical position, with offsets of approximately 1250 bp and 340 bp, respectively. The sgRNA was then tested on its ability to cut the double-stranded DNA amplicons in the presence of Cas9 Nuclease according to New England BioLab’s protocol “In vitro digestion of DNA with Cas9 Nuclease, S. pyogenes (M0386).” The negative control did not contain the sgRNA for T/brachyury. The reactions consisted of: 30 µl nuclease-free water, 4.5 µl NEBuffer r3.1, 1.5 µl 1 µM Cas9 Nuclease, S. pyogenes (M0386), and 4.5 µl 300 nM T sgRNA or 4.5 µl nuclease-free water, respectively, and were incubated for 10 min at 25 °C, after which 4.5 µl of 30 nM T amplicons were added for a total reaction volume of 45 µl and further incubation for 30 min. The reactions were stopped by heating the samples to 65 °C for 5 min, then the DNA was ethanol precipitated, pellets were resuspended in TE buffer, mixed with Gel Loading Dye, Purple (6X) (NEB, B7024S), and resolved on a 1.1% agarose gel for separation of any fragments produced by the ribonucleoprotein complex (RNP). GelRed (Biotium) staining served to visualize DNA, and images were acquired with a BioRad Transluminator (Universal Hood II).

### Mice

All mice utilized for this study were housed and maintained at Pennington Biomedical Research Center under specific pathogen-free (SPF) conditions, in individually vented cages.

Females of the inbred FVB/N mouse strain (Charles River Laboratories International, Inc.) were used as zygote donors, chosen for their breeding success, high oocyte yield and excellent microscopic visibility of fertilized oocyte pronuclei^36^. These mice had a light cycle from 7 am to 7 pm and were superovulated before being mated. For superovulation, 5 IU of Pregnant Mare Serum Gonadotropin (PMSG, ProSpec-Tany TechnoGene Ltd) were injected intraperitoneally one hour past the middle of the light cycle two days before breeding, and 5 IU Human Chorionic Gonadotropin (hCG, ProSpec-Tany TechnoGene Ltd) were injected at 11am, approximately 1 h before mating was set up with a FVB/N male. The day of observation of a copulation plug was counted as gestational day E0.5. Females of the outbred CD-1 mouse strain (Crl:CD1(ICR), Charles River Laboratories International, Inc.) were used as surrogates, after mating with a vasectomized CD-1 male to induce pseudopregnancy. The female foster dams were used at > than 6 weeks of age, they weighed between 25 and 32 g, and they experienced the same light cycle as the donor mice.

### Microinjection and transfer of zygotes

Single-cell zygotes were harvested from plugged FVB/N donors on E0.5 and were cultured in EmbryoMax® Advanced KSOM Embryo Medium (EMD Millipore) at 37 °C in an atmosphere containing 5% CO_2_ before and after microinjection. Reagents to be microinjected were mixed fresh 30 min before injection and consisted of 25 ng/µl TrueGuide™ sgRNA for the gene of interest (T/brachyury) and 50 ng/µl TrueCut™ Cas9 Protein v2 (Invitrogen) diluted in EmbryoMax® Electroporation Buffer (EMD Millipore). The injection solution was stored on ice until use. Microinjections and transfer surgeries were performed as described by Behringer et al. in “Manipulating the Mouse Embryo”^37^. A FemtoJet 4i Microinjector (Eppendorf) was employed for injections. Injected embryos were transplanted to the oviduct of pseudopregnant CD-1 females. A maximum of 18 injected zygotes were transferred into the infundibulum of a single uterine horn. Noon of the day of the embryo transfer transplantation surgery was designated day E0.5 of gestation. Surrogate females were allowed to carry until E9.5, when they were euthanized for embryo harvest.

### Dissection, imaging, and fixation of embryos

Dissections of individual decidua were performed each in their own fresh dish of OptiMEM, and then the yolk sac and any extraembryonic tissues destined for genotyping were rinsed in separate wells of PBS in a 24-well plate. Embryos were imaged fresh in a plate of 5% FBS Opti-MEM® I Reduced Serum Medium (Life Technologies) under a Leica S9i stereo microscope with V4.12.0 Leica Application Suite software (Leica Microsystems Co.). Thereafter, the embryos were rinsed in ice-cold sterile PBS and fixed in 4% paraformaldehyde + 0.25% glutaraldehyde at 4 °C for 3 h. Fixed embryos were then dehydrated in methanol washes and stored at − 20 °C.

### Isolation of genomic DNA for genotyping

For genotyping of genome editing events at the T/brachyury locus, visceral yolk sacs/visceral endoderm, chorion, and allantois (where available) were harvested from each embryo. These tissues were rinsed in sterile PBS buffer, stored individually in 50 μl PBS in microtubes, and frozen at − 20 °C until genomic DNA (gDNA) isolation by the Tail buffer/Proteinase K digestion protocol^38^.

### PCR amplification for sequencing

A fragment of 6572 bp size was PCR amplified from genomic DNA with approximately 3 kb on either side flanking the targeted cut site ϴ. Amplifications were performed in 50 μl volume, with 50 ng of template, 200 nM primers, 250 μM each dNTP and 1.0 U Phusion High-Fidelity DNA Polymerase (New England BioLabs). Primer pairs for T CRISPants were T-Forward TCACCGAGAGGCAATAAACC and T-Reverse GCTGGCGTTATGACTCACAG (Chromosome 17: 8,433,450–8,433,470, and Chromosome 17: 8,440,002–8,440,022, respectively; ENSEMBL Gene ID: ENSMUSG00000062327 GRCm38.p6). Amplification conditions were: Hotstart, with 1.5 min at 98 °C, followed by 28 cycles of 10 s melting at 98 °C, 30 s annealing at 67 °C, 6 min extension at 72 °C, with a final step of 10 min at 72 °C, and storage at 4 °C thereafter. When necessary, the reaction was performed twice to yield sufficient amounts of amplicon. Electrophoresis was employed to resolve the amplified products on 1% agarose gels with the 1 kb Plus DNA Ladder (Invitrogen) as size marker. GelRed (Biotium) staining was used to visualize DNA, and images were acquired with a BioRad Transluminator (Universal Hood II). After confirmation of a PCR product, the amplicons were purified with the QIAquick PCR Purification Kit (Qiagen) according to the manufacturer’s protocol, and subsequently prepared for sequencing via Oxford Nanopore Technologies’ MinION Portable Sequencer.

### Preparation of libraries for the MinION portable sequencer

Purified PCR amplicons were barcoded using LongAmp Taq 2 × Master Mix (New England BioLabs) and Oxford Nanopore Technologies (ONT) PCR Barcoding Expansion Pack 1–96 (EXP-PBC096) according to ONT’s PCR barcoding (96) amplicons protocol. Barcoding primers were 24 bp in length, added to both ends. Sequencing adaptors were added using the 1D-Ligation Sequencing Kit (SQK-LSK109) instructions (ONT). At all steps, DNA was purified using AMPure XP beads (Agencourt) with a 1 × beads to sample ratio and quantified with a Qubit fluorometer. Up to 50 fmol of sequencing libraries were loaded on primed FLO-MIN106 flow cells (R9.4.1) (ONT). Runs were performed employing the ONT MinION sequencer and MinKNOW GUI at Kit SQK-LSK109, Exp-PBC096 barcoding, and High-accuracy basecalling settings for up to 24 h (ONT). The MinKNOW GUI and EPI2ME software demultiplexed and trimmed barcodes for each sample, placing fast5 and fastq reads in barcode-specific labelled folders. On average, close to 5000 sequence reads per sample were obtained; for samples yielding under 2000 reads, a new sequencing run was conducted.

### Analysis of ONT sequencing data

De-multiplexed fastq reads were filtered via SeqKit^39^ with the stipulation that all reads to be analyzed had to contain at least 250 bases from each end of the amplicon, simultaneously, collecting only complete reads for further analysis. After high accuracy base-calling, the CRISPResso2 software pipeline^40^ was used to analyze genome editing of the amplicon sequences by aligning sequences wildtype T locus control samples run alongside the experimental samples, and to the reference genome sequence for the T/Brachyury gene. This pipeline quantifies insertions, mutations, and deletions to determine whether a read is modified or unmodified and then summarizes genome editing results in plots and datasets^41^. First, input reads were filtered according to quality score (phred33), by which potentially false-positive indels were removed. Amplicon minimum alignment score to the target amplicon was set at 60. For samples with deletions larger than 1 kb, this score was lowered to 45 (denoted in the command "-amas 45") and the Gap open option for Needleman-Wunsch alignment was changed from the default of − 20 to − 30 (denoted in the command "-needleman_wunsch_gap_open-30") to allow the pipeline to assess reads containing these large deletions.

### Research involving laboratory animals

All animal experimentation protocols in this study were reviewed and approved by the Institutional Animal Care and Use Committee (IACUC) at Pennington Biomedical Research Center/Louisiana State University System. All procedures involving mice were compliant with the "Guide for Care and Use of Animals in Laboratory Research" (https://pubmed.ncbi.nlm.nih.gov/21595115/; 10.17226/12910), as required by the US National Institutes of Health. The currently active protocol #1044 was approved by the Pennington Biomedical Research Center IACUC for the epriod from December 18, 2018 through December 17, 2021. Experimentation and analysis were performed following ARRIVE (Animal Research: Reporting In Vivo Experiments) guidelines (https://arriveguidelines.org/arrive-guidelines), which were also applied in the writing of this manuscript.

### Research involving recombinant DNA

All experiments involving the production and purification of recombinant DNA were reviewed and approved by the Institutional Biological Safety Committee at Pennington Biomedical Research Center/Louisiana State University System.

## Data Availability

The datasets generated and analyzed during the current study are available from the corresponding author on reasonable request, in compliance with institutional policies of Pennington Biomedical Research Center/Louisiana State University System.
